# Obstructed Hemivagina and Ipsilateral Renal Anomaly Syndrome Rare Obstructive Uterovaginal Anomaly: A Case Report

**DOI:** 10.31729/jnma.5043

**Published:** 2020-10-31

**Authors:** Tulasa Basnet, Tarun Pradhan, Punita Yadav, Manoj Sah, Jyotsna Yadav, Yashaswi Rai, Rashmi Thapa

**Affiliations:** 1 Department of Obstetrics and Gynaecology, B. P. Koirala Institute of Health Sciences, Dharan, Nepal

**Keywords:** *ipsilateral renal anomaly*, *paravaginal mass*, *uterovaginal anomaly*

## Abstract

Obstructed Hemivagina and Ipsilateral Renal Anomaly syndrome is a rare obstructive uterovaginal anomaly involving both mesonephric and paramesonephric ducts. It usually presents after menarche with non-specific symptoms like pelvic pain, dysmenorrhea, or paravaginal mass and examination findings of paravaginal or pelvic mass. Because of non-specific symptoms and signs, the diagnosis is usually overlooked, which leads to complications like endometriosis, tubo-ovarian abscess compromising patient's fertility, and quality of life. Therefore, in presence of these nonspecific clinical features along with imaging findings of uterine didelphys and unilateral renal agenesis, this syndrome should be considered in the diagnosis. We present a case of a 17-year-old lady with Obstructed Hemivagina and Ipsilateral Renal Anomaly syndrome, diagnosed by finding of paravaginal mass on examination and uterine didelphys with ipsilateral renal agenesis in USG and managed successfully by resection of vaginal septum.

## INTRODUCTION

Obstructed Hemivagina and Ipsilateral Renal Anomaly (OHVIRA), also known as Herlyn-Werner-Wunderlich (HWW) syndrome is a rare combined paramesonephric and mesonephric duct anomaly.^1^ The syndrome is characterized by the presence of uterine didelphys, blind hemivagina, and renal anomalies, the most common renal anomaly being renal agenesis.^2^ OHVIRA constitutes 2-3% of all Mullerian anomalies.^3^ This condition usually presents after menarche,typically presentswith non-specific symptoms of pelvic pain or dysmenorrhea and a paravaginal bulge on examination in an adolescent girl. However, it can also present with an acute abdomen and tubo-ovarian mass.^1,4^ The nonspecific presenting complaints and heterogeneity of presentation can make the diagnosis a challenge.

## CASE REPORT

**Figure 1.**
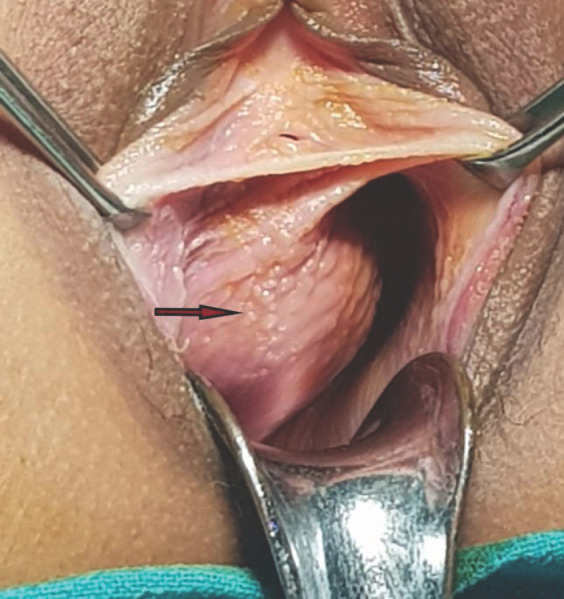
Paravaginal mass on right side of vagina.

A 17-year-old married P_0_A_1_ presented to the OBGYN outpatient department with complaints of mass associated with pain at vaginal introitus for the last one year. She also complained of difficulty in passing urine with a small urinary stream, intermittent flow, and a sense of incomplete evacuation. Her menstrual cycles were irregularly occurring every 30-45 days since her menarche at 14 years of age but did not have menorrhagia or dysmenorrhea. She did not have dyspareunia but complained of some discomfort during coitus because of the mass. She was married for one year and had an induced medical abortion at six weeks of gestation for unwanted pregnancy 8 months back. She had then been regularly taking low dose combined oral contraceptive pills. On examination, a cystic, nonreducible bulge of around 5X7 cm was seen on the right lateral vaginal wall extending up to the right fornix which was non-tender on palpation. On per speculum examination, the cervix was visualized and on bimanual examination,the uterus was deviated to the right side and was 10 weeks size. A provisional diagnosis of right lateral vaginal wall cyst was made and she was advised ultrasonography to rule out other pelvic pathologies (Figure 1).

**Figure 2A.**
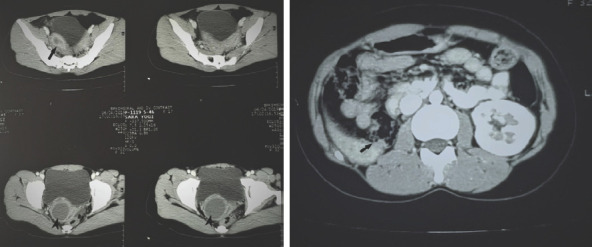
Two widely separated endometrial myometrial complexes with mild fluid collection in the right endometrial cavity (arrow) and fluid collection in right hemivagina (arrowheads), 2B. Absent right kidney (arrow).

Her urine routine and microscopy examination were normal and urine culture was sterile. Ultrasonography showed uterine didelphys with mild fluid collection in the right endometrial cavity with fluid collection distending vagina on the right side i.e. unilateral hematocolpos and non-visualization of the right kidney. Although the MRI pelvis was planned next, it was not afforded by the patient. Instead, she followed up with a CT scan of the pelvis which showed two widely separated endometrial myometrial complexes with two cornua, body, and cervix of the uterus (uterine didelphys) with mild fluid collection in the right endometrial cavity and vagina on the right side (unilateral hematometrocolpos) (Figure 2A). There was a minimal fluid collection in the left endometrial cavity. The right kidney was not visualized (Figure 2B). All findings were consistent with Obstructed Hemivagina and Ipsilateral Renal Anomaly (OHVIRA) syndrome.

**Figure 3A.**
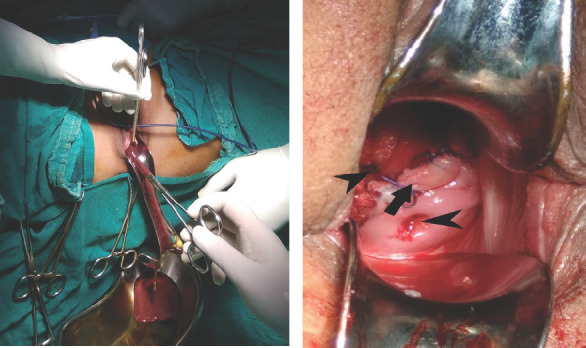
Drainage of collected blood, 3B. Two cervices (arrowheads) visible after resection of vaginal septum (arrow).

She and her husband were counseled regarding the condition and management plan. After all preoperative investigations and evaluation, drainage of hematometrocolpos, and resection of the vaginal septum was performed as a single-step procedure. Preoperative antibiotic prophylaxis was given. The vaginal pouch was opened and around 150 ml of inspissated blood was drained (Figure 3A). Complete resection of the septum was performed and the raw edges were sutured for hemostasis (Figure 3B).

Her postoperative period was uneventful. She was discharged on the third postoperative day. She followed up after 15 days in the OPD when she reported no symptoms, her per speculum examination revealed two cervices. She was counseled regarding the possibility of vaginal stenosis. She followed up again after her next menstrual period. On per speculum examination, the vagina looked normal and both cervices were visualized. She was reassured of the normal findings and asked to follow up if she had any problems.

## DISCUSSION

The incidence of obstructed hemivagina and ipsilateral renal anomaly (OHVIRA), is reported to be 2-3% of all Mullerian anomalies in various case series.^3^ Other studies have reported its proportion to be between 0.16 to 10% of all Mullerian anomalies.^5^ The condition was first reported by Purslow in 1922.^6^ For the first 60 years after the first reported case, only 115 cases were reported.^4^ Poor awareness and incorrect diagnosis could have been responsible for the under-reporting. However, in recent years multiple large case series have been published.^1,4,6,7^ This may be because of the increasing awareness about the condition as well as improvement in diagnostic modalities.

The frequent association of genital anomalies with urinary system anomalies is because the development of the genital and urinary system is closely related. The mesonephric ducts, not only give origin to the ipsilateral renal system but also act as inductor elements for the adequate fusion of Mullerian ducts. The ureteric bud arises from the opening of the mesonephric duct in the urogenital sinus which is responsible for the formation of the kidney on that side. In the absence of a mesonephric duct on one side, the ipsilateral kidney and ureter fail to develop. The ipsilateral Mullerian duct is displaced laterally which then fails to fuse adequately with the urogenital sinus, leading to the formation of a blind sac, or obstructed hemi-vagina. The latter also fails to fuse with the contralateral mOllerian duct which results in uterine didelphys.^6,8,9^ Though the commonly reported uterine anomaly is uterine didelphys, other anomalies like septate and bicornuate uterus also have been reported. Meanwhile, the distal part of the vagina which arises from the urogenital sinus is not affected and develops normally. Similarly, commonly reported unilateral genital obstruction is vaginal, but cervical obstruction or atresia has also been reported. In the case of renal malformations, the commonest anomaly is ipsilateral renal agenesis; however, duplicated and dysplastic kidneys have also occasionally been reported.^6^

In literature, both obstructed hemivagina and renal agenesis have been reported slightly more often on the right side (around 60%) in OHVIRA.^1,6,10^ Our patient also had obstructed right hemivagina and right renal agenesis with uterine didelphys.

Patients with OHVIRA syndrome are usually asymptomatic till puberty. Diagnosis is usually made after menarche with most patients diagnosed within 2 years after menarche due to the development of hematocolpos or hematometra. Some may even present in early infancy with mucocolpos or hydrocolpos due to secretions in obstructed vagina under the influence of maternal hormones.^11^ In one review from China in 2013, the reported median duration from menarche to diagnosis was one year (range: 0-25 years).^1^ Thus, cases may present quite late in reproductive life like our patient who presented at the age of 17 years, three years after her menarche. A case series from the USA in 2007 found the median age of presentation as 14 years with a range of 10-29 years^4^ while another series had mean age 20.7 years with a range of 11-42 years.^6^ Age of presentation also varied according to whether the vaginal obstruction was complete or incomplete i.e. 12.8±1.84 years in complete obstruction and 20.68±7.43 years in incomplete obstruction.^1^

The common clinical presentation of the condition has remained the same over the years since the first reported case. However, increasing experience has broadened the understanding of the heterogeneity of the anatomic disorders among clinicians. An adolescent girl with nonspecific symptoms of pelvic pain or dysmenorrhea is described as a typical presentation of this condition. On examination, the finding of a visible vaginal sidewall bulge leads to the diagnosis of an obstructed hemivagina, and also the finding of a second cervix.^4^ Common presenting symptoms are pelvic pain, dysmenorrhea, foul-smelling discharge, and pelvic mass or paravaginal mass.^1,2,12^ Our patient did not have symptoms like dysmenorrhea or cyclical abdominal pain but presented with paravaginal mass and difficulty in micturition. Periodicity of menstrual cycles is usually unaffected in this syndrome, as in our case. However, irregular vaginal bleeding has been reported in 20-25% of patients. Some other cases presenting with complications of obstructed hemivagina like endometriosis and tubo-ovarian abscess have also been reported .^1,4^

Early detection and management are necessary to prevent associated complications like infertility, endometriosis, pelvic adhesions, and pyosalpinx or pyocolpos. Because of non-specific symptoms and lack of menstrual irregularity, the possibility of Mullerian anomaly in these patients is easily overlooked. Since the vagina is quite distensible and can accommodate a large amount of accumulated blood on the obstructed side, these patients can go unrecognized for years. Also, there is sufficient absorption of menstrual blood in between periods.^13^ Proper history-taking, detailed physical examination, and appropriate imaging modalities help reach the correct diagnosis. In our case, just from history and examination, diagnosis of OHVIRA syndrome was not thought of. Ultrasonography was advised to see the extent of paravaginal mass and also to rule out other pelvic pathology. However, it led us to the diagnosis.

Both Ultrasonography and Magnetic resonance imaging (MRI) is helpful in diagnosis as well as in planning management of the case. While ultrasonography can provide information about uterovaginal duplication, haematocolpos or haematometrocolpos, and the absence of the ipsilateral kidney, MRI pelvis provides more detailed information regarding uterine morphology, its continuity with each vaginal channel, and the nature of the uterovaginal contents.^14^ The role of computed tomography (CT) is limited due to radiation exposure and limited soft-tissue resolution. It does not usually add extra information on sonography findings.^4,8^ Our patient underwent a CT scan of the abdomen and pelvis which confirmed the USG findings but did not add any further information. The main objective of the surgical management of this condition is to relieve obstruction and prevent associated complications. Treatment includes full resection of the vaginal septum. Resection of as much of septal tissue as possible with special care to avoid both cervices bladder and rectum should be made. Many times obstructed cervix is dilated and pulled towards the septum medially.^15^ Because of the anatomic distortion from hematocolpos and chronic inflammation as well as the narrow vagina in adolescent girls, the procedure becomes difficult. Therefore, some prefer a two-stage procedure with a reduction of hematocolpos in the first stage and resection of the septum in the second stage.^4^ However, in our case we performed a single-stage procedure. The use of laparoscopy to assess exact uterine anatomy at the time of resection of the vaginal septum has also been advocated; however, its effect on prognosis is not yet known.^16^ Vaginal stenosis may occur post-operatively. We followed up with our patient after she had her menstrual cycle two months later. On examination, two cervices were seen and there was no stricture.

OHVIRA syndrome is a rare condition and many treating doctors being unfamiliar with the condition, it does not come as a common diagnosis in clinical practice. And also this condition does not always present with specific symptoms. Therefore, a high degree of suspicion, proper knowledge of the condition, and use of appropriate imaging techniques is a must for the diagnosis. Early diagnosis and appropriate treatment area must for relieving symptoms, preventing complications related to obstruction, and preserving reproductive function.

## Consent:

**JNMA Case Report Consent Form** was signed by the patient and the original article is attached with the patient's chart.

## Conflict of Interest

**None.**
